# Estudio Parto: postpartum diabetes prevention program for hispanic women with abnormal glucose tolerance in pregnancy: a randomised controlled trial – study protocol

**DOI:** 10.1186/1471-2393-14-100

**Published:** 2014-03-10

**Authors:** Lisa Chasan-Taber, Bess H Marcus, Milagros C Rosal, Katherine L Tucker, Sheri J Hartman, Penelope Pekow, Barry Braun, Tiffany A Moore Simas, Caren G Solomon, JoAnn E Manson, Glenn Markenson

**Affiliations:** 1 Division of Biostatistics & Epidemiology, Department of Public Health, School of Public Health & Health Sciences, University of Massachusetts, 405 Arnold House, 715 North Pleasant Street, Amherst, MA 01003-9304, USA; 2 Department of Family and Preventive Medicine, School of Medicine, University of California San Diego, La Jolla CA, USA; 3 Division of Preventive and Behavioral Medicine, Department of Medicine, University of Massachusetts Medical School, Worcester MA, USA; 4 Department of Kinesiology, School of Public Health & Health Sciences, University of Massachusetts, Amherst MA, USA; 5 Departments of Obstetrics & Gynecology and Pediatrics, University of Massachusetts Medical School, Worcester MA, USA; 6 Brigham and Women’s Hospital and Harvard Medical School, Boston MA, USA; 7 Baystate Medical Center, Springfield MA, USA

**Keywords:** Lifestyle intervention, Randomised controlled trial, Healthy eating, Prevention, Diet, Latina, Physical activity, Postpartum, Pregnancy, Gestational diabetes mellitus, Transtheoretical model

## Abstract

**Background:**

Diabetes and obesity have reached epidemic proportions in the U.S. with rates consistently higher among Hispanics as compared to non-Hispanic whites. Among Hispanic women diagnosed with gestational diabetes mellitus (GDM), 50% will go on to develop type 2 diabetes within 5 years of the index pregnancy. Although randomised controlled trials among adults with impaired glucose tolerance have shown that diet and physical activity reduce the risk of type 2 diabetes, such programs have not been tested in high-risk postpartum women. The overall goal of this randomised controlled trial is to test the efficacy of a culturally and linguistically modified, individually-tailored lifestyle intervention to reduce risk factors for type 2 diabetes and cardiovascular disease among postpartum Hispanic women with a history of abnormal glucose tolerance during pregnancy.

**Methods/Design:**

Hispanic pregnant women who screen positive for GDM will be recruited and randomly assigned to a Lifestyle Intervention (n = 150) or a Health & Wellness (control) Intervention (n = 150). Multimodal contacts (i.e., in-person, telephone, and mailed materials) will be used to deliver the intervention from late pregnancy (29 weeks gestation) to 12 months postpartum. Targets of the intervention are to achieve Institute of Medicine Guidelines for postpartum weight loss; American Congress of Obstetrician and Gynecologist guidelines for physical activity; and American Diabetes Association guidelines for diet. The intervention draws from Social Cognitive Theory and the Transtheoretical Model and addresses the specific cultural and environmental challenges faced by low-income Hispanic women. Assessments will be conducted at enrollment, and at 6-weeks, 6-months, and 12-months postpartum by trained bicultural and bilingual personnel blinded to the intervention arm. Efficacy will be assessed via postpartum weight loss and biomarkers of insulin resistance and cardiovascular risk. Changes in physical activity and diet will be measured via 7-day actigraph data and three unannounced 24-hour dietary recalls at each assessment time period.

**Discussion:**

Hispanic women are the fastest growing minority group in the U.S. and have the highest rates of sedentary behavior and postpartum diabetes after a diagnosis of GDM. This randomised trial uses a high-reach, low-cost strategy that can readily be translated into clinical practice in underserved and minority populations.

**Trial registration:**

NCT01679210

## Background

Type 2 diabetes is a global epidemic affecting approximately 21 million people in the U.S. [1]. The age at onset for type 2 diabetes has decreased [1], highlighting the importance of identifying high-risk groups early, in order to implement prevention efforts. One such high-risk group is women who develop glucose intolerance during pregnancy. Both gestational diabetes mellitus (GDM) and milder glucose intolerance in pregnancy identify women who are at high risk for type 2 diabetes [2,3]. Indeed, a recent meta-analysis found that GDM confers a 7-fold risk for future type 2 diabetes [4] and up to one third of women with type 2 diabetes have previously been diagnosed with GDM [5].

According to a recent systematic review, the highest risk period for the development of type 2 diabetes is within the first 5 years after a GDM pregnancy [2], with 50% of Hispanic women developing type 2 diabetes within 5 years [6]. This is consistent with recent findings showing a rapid postpartum change in glucose tolerance [7]; by 12 months postpartum, 17.1% of those with recent GDM and 10% of women with milder degrees of gestational glucose intolerance had progressed to prediabetes or diabetes [8]. In other words, pregnancy unveils a preexisting susceptibility for type 2 diabetes and offers the opportunity to implement interventions to decrease such risk.

In spite of these observations, studies of diabetes prevention among high-risk postpartum women with history of GDM are sparse. In the Diabetes Prevention Program (DPP) [9] women with a history of GDM, approximately a decade after their pregnancy, were able to decrease their diabetes risk with a weight reduction goal of 7% of their baseline weight even though they lost less weight than the overall DPP sample (1.60?±?0.80 kg vs. 4.03?±?0.40 kg at 3 years, p?=?0.021) [10]. This weight loss was achieved through behavioral goals of ≥150 min per week of physical activity and a low-fat, low-calorie diet. However, this trial involved an intensive intervention not easily administered in a clinical setting and was conducted an average of 12 years after GDM diagnosis such that women with early postpartum conversion to diabetes, and therefore at highest risk, were not eligible. Other limitations included intervening lifestyle factors and subsequent pregnancies which may have modified findings, reliance on self-reported diagnosis of GDM, lack of laboratory measures on glucose tolerance during pregnancy, as well as a small percentage of Hispanic women.

Weight loss can be achieved in the immediate postpartum period [11], with studies finding that both energy intake restriction and physical activity are needed to reduce weight [12,13], compared to exercise alone [14]. Similarly, weight loss interventions among non-pregnant adults [15-17] have shown that multifaceted interventions compared with stand-alone dietary advice, exercise modification, or behavioral strategies yield significant improvements in health outcomes and weight loss. A recent review calculated that lifestyle interventions for women with a history of GDM have the potential to delay or prevent one-sixth of type 2 diabetes cases in the female population [18].

Hispanics are the largest minority group in the U.S., with the highest birth and immigration rates of any minority group [19]. It is estimated that by 2050, Hispanic women will comprise 30.2% of the female population in the United States [20]. Hispanics are the most physically inactive ethnic group in the U.S. [21] and are disproportionately affected by overweight and obesity. At each BMI level, Hispanics have a higher prevalence of diabetes than non-Hispanic whites [22-24]. Furthermore, a significant proportion of Hispanics lack awareness of diabetes risk factors and prevention strategies [25]. Despite the high rates of progression of GDM to type 2 diabetes, only one in five Hispanic women receive recommended postpartum diabetes screening, the lowest follow-up frequency for any group [26]. Due to cultural factors, socioeconomic circumstances, differences in educational background, and language barriers, Hispanics have had limited access to clinical and public health interventions that promote healthy lifestyles. Therefore, the overall objective of this trial is to test the ability of a lifestyle intervention, informed by formative behavioral research, to reduce risk factors for type 2 diabetes and cardiovascular disease among postpartum Hispanic women with a recent history of abnormal glucose tolerance (AGT) during pregnancy.

### Aims

Specific aims are to achieve the Institute of Medicine (IOM) guidelines for postpartum weight loss and to improve maternal metabolic status by achieving and maintaining 1) postpartum weight reduction to prepregnancy weight if prepregnancy BMI was in the normal range, or to achieve a 5% reduction from prepregnancy weight if prepregnancy BMI was overweight/obese [27]; 2) at least 150 min per week of moderate intensity physical activity such as brisk walking, as recommended by ACOG for the postpartum period [28]; and 3) reduction in postpartum total energy intake via reduced consumption of popular energy-dense foods (e.g., fast food, high-fat snacks, fried foods and sugar-sweetened beverages), reduced portion size, appropriate modifications in ethnic recipes, and higher fruit and vegetable intake, as recommended by the ADA [29].

## Methods

**Figure 1:**
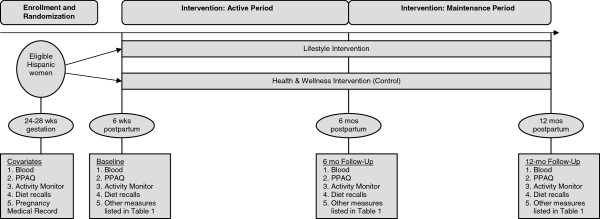
Trial design.

Estudio Parto (Project Aiming to Reduce Type twO diabetes) will be based at the ambulatory obstetrical practices of Baystate Medical Center in Western Massachusetts. Eligible pregnant Hispanic women who screen positive for GDM will be recruited between 24-28 weeks gestation and randomly assigned to a Lifestyle Intervention (n = 150) or a Health & Wellness (control) Intervention (n = 150). Multimodal contacts (i.e., in-person, telephone counseling, and mailed print-based materials) will be used to deliver the intervention from late pregnancy (29 weeks gestation) to 12 months postpartum, encompassing introductory, active, and maintenance phases (Figure 1).

### Eligibility criteria

Baystate Medical Center practices universal screening for GDM, which consists of a random 50-g glucose load and a plasma glucose determination 1 hour later. If the plasma glucose value is ≥135 mg/dL, a diagnostic 100-g 3 hour oral glucose tolerance test (OGTT) is conducted. Eligible participants will be pregnant Hispanic women who screen positive for GDM (50-g glucose load ≥135 mg/dL). We will exclude women with: 1) history of type 1 or type 2 diabetes, heart disease, or chronic renal disease, 2) contraindications to postpartum participation in the trial’s intervention activities which include engagement in moderate physical activity and consumption of a low-fat/high-fiber diet (e.g., Crohn’s disease, ulcerative colitis), 3) inability to read English or Spanish at a 6th grade level, or 4) <18 or >45 years of age.

### Recruitment

The trial will capitalize on our expertise in culturally appropriate strategies for recruiting pregnant Hispanic women in practice-based settings [30]. Specifically, bilingual (Spanish and English) and bicultural health educators will recruit women at the time of routine GDM screening. Women will be informed of the aims and procedures of the project and will be asked to sign a written informed consent form, as approved by the Institutional Review Boards of the University of Massachusetts-Amherst and Baystate Health. Upon enrollment, the baseline assessment will be conducted which includes: 1) data collection of behaviors during pregnancy via standardized questionnaires, 2) measures of physical activity via an ActiGraph GT3X-plus activity monitor (Actigraph LLC, Pensacola, FL) to be worn on the wrist for the next 7 days, 3) measures of dietary intake via three unannounced 24-hr dietary recalls over the following two-week period, 4) laboratory measures of biomarkers of insulin resistance and cardiovascular risk via a study-specific fasting blood sample. Completion of each assessment and blood draw is followed by a gift card.

### Randomisation

Randomisation to the Lifestyle Intervention or to the Health & Wellness (control) Intervention will occur at ~29 weeks gestation, after completion of the baseline assessment. Randomisation will be stratified based on the results of the diagnostic 100-g OGTT using thresholds defined by the American Diabetes Association (ADA) [31] (≥ 95 mg/dL fasting and?≥?180 mg/dL, ≥155 mg/dL and ≥140 mg/dL at 1 hour, 2 hours and 3 hours, respectively): 1) no glucose values meeting or exceeding the ADA thresholds; or 2) one or more glucose values meeting or exceeding the ADA thresholds.

### Lifestyle intervention

The Lifestyle Intervention is an evidence-based approach utilizing culturally and linguistically modified, motivationally targeted, individually-tailored intervention materials developed in our prior randomised controlled trials among Hispanics [32-39]. The intervention draws from Social Cognitive Theory [40] and the Transtheoretical Model [41] and takes into account findings by our research group on the specific social, cultural, economic, and environmental resources as well as challenges faced by women of Hispanic backgrounds [25,42,43].

The Introductory Phase (~29 weeks gestation - delivery) (Figure 1) will start with a face-to-face session. The goal of this phase is to optimize gestational weight gain for the remaining pregnancy period and move women over the continuum of pre-contemplation to contemplation and preparation for the postpartum Active Intervention Phase. Motivational interviewing principles will be used to identify and strengthen women’s motivations for change. Specifically, the session will target knowledge and attitudes regarding gestational weight gain, postpartum weight loss, and type 2 diabetes prevention. Gestational weight gain guidelines will be reviewed and participants will be provided with a digital scale.

The session will additionally include administration of diet and physical activity questionnaires that facilitate tailoring the intervention. The Diet Tailoring Questionnaire is a two-part survey consisting of a checklist of high-calorie and lower-calorie foods and beverages commonly consumed by the target population. Respondents are asked to indicate the frequency that they consume the various foods and rate their confidence (self-efficacy) in their ability to decrease high calorie and increase low calorie food consumption. The Exercise Tailoring Questionnaire consists of 3 measures: Stages of Change for Physical Activity [44], Processes of Change for Physical Activity [45], and Self-Efficacy for Physical Activity [44].

This session will be followed by a third trimester booster telephone counseling session which will review progress toward gestational weight gain management and reinforce preparation for postpartum weight loss. Print-based intervention materials, in Spanish or English depending upon participant preference and written at a 6th grade reading level, will be mailed.

The Active Phase (6 weeks postpartum – 6 months postpartum) (Figure 1) will start with a face-to-face session. The tailoring questionnaires will be repeated and counseling will include the development of individualized weight loss, physical activity, and dietary goals. This session will be followed by weekly, biweekly, and monthly mailed, print-based materials as well as telephone booster calls to provide motivationally-based individualized feedback. Specifically, mailed materials are culturally modified, individually-tailored and motivationally targeted based on responses to monthly mailed tailoring questionnaires. Booster telephone sessions facilitate: 1) review of progress toward dietary, physical activity and weight loss goals, 2) problem-solving of challenges faced in achieving goals (e.g., balancing caregiver/household responsibilities, cultural norms of self-sacrifice, limited social support, partner negotiation, and neighborhood safety), 3) discussion of mailed tip sheets, and 4) assistance with setting new goals.

The Maintenance Phase (6 – 12 months postpartum) (Figure 1) will involve continued telephone sessions and mailed materials on a monthly and then bimonthly schedule.

### Curriculum

At the outset of the Active Phase of the intervention, a weight-loss goal based on prepregnancy BMI will be set and participants will be encouraged to work toward this goal by focusing on a reduction of 1-2 pounds per week. The health educator will utilize a checklist of motivators for wanting to lose weight, specific to new mothers, to help women identify their own weight loss motivations and to reinforce engagement. Participants will be encouraged to weigh themselves at home daily and to chart their weight on a grid weekly.

Physical activity change will be targeted via individualized week-by-week goals that focus on increasing the time spent in moderate intensity activity, as well as steps taken per day.

Women will choose what form of safe activity they enjoy the most or can most readily fit into their lifestyle, from dancing to walking in a shopping mall to yard work. The accumulation of short bouts (i.e., 10 min episodes) will be encouraged. Pedometers and activity logs will be provided for women to monitor their activity. Based on responses to the tailoring questionnaires, individually-tailored computer Expert-System Feedback Reports [37,39,46,47] will draw particular messages from a library of approximately 296 messages regarding motivation, self-efficacy, and cognitive and behavioral strategies for exercise adoption. Stage-Matched Manuals will focus on the benefits of exercise, building social support for new behavioral patterns, and strategies for overcoming barriers to exercise specific to low-income Hispanic women. Tip Sheets include topics such as stretching and exercising with baby (e.g., walking while pushing a stroller).

Dietary change will be targeted by working toward an ultimate goal of 1,500 calories per day (up to 2,000 for breastfeeding women who lose more than 2 lbs/week over a 2 week period). Dietary goals will be tailored to the participant based on responses to the Diet Tailoring Questionnaires, which take into consideration energy-dense foods that the participant eats frequently and feels confident that she is able to reduce or replace, and lower calorie foods which the participant eats infrequently and feels confident that she can increase. Participants will be instructed in how to self-monitor dietary intake using the food calorie guide and a tip sheet for measuring portion sizes. Participants will be provided with measuring cups and a dietary log.

Quality control procedures will ensure that stage of change and social cognitive constructs are consistently represented in all intervention materials.

### Health & wellness (control) intervention

To ensure retention and to control for contact time, the Health & Wellness (control) arm will receive mailed health materials and telephone booster calls on the same schedule as the Lifestyle Intervention arm. Mailed materials focus on non-exercise and non-dietary topics and include booklets from ACOG and the American Academy of Pediatrics in English or Spanish. These booklets represent high-quality, standard, low-cost, self-help material currently available to the public. In this way, we control for contact time, while keeping the content of the two interventions distinct. Hispanic control-arm participants in our prior studies reported that these materials were of interest and differential dropout did not occur between study arms [36,48].

### Outcome variables

Postpartum weight loss will be measured as the difference between weight at 6 or 12 months postpartum and weight at delivery, and will be calculated as absolute weight change according to prepregnancy BMI, percentage who retain a specific amount of weight over prepregnancy weight, or proportion whose BMI category changes from prepregnancy BMI category as have others (Table 1) [12-14].

**Table 1:** Variables collected at assessment time points

	**Pregnancy**	**Postpartum**		
	**3rd trimester**	**6 wks**	**6 mos**	**12 mos**
**Aim #1: Postpartum weight loss**	X	X	X	X
**Aim #2: Postpartum biomarkers of insulin resistance**				
Glucose	X	X	X	X
Insulin	X	X	X	X
HbA1c	X	X	X	X
Adiponectin	X	X	X	X
Leptin	X	X	X	X
TNF-α	X	X	X	X
HOMA	X	X	X	X
Area-Under-The-Glucose Curve (AUC)	X	X	X	X
**Aim #3: Other postpartum biomarkers of cardiovascular risk**				
Total cholesterol	X	X	X	X
Triglycerides	X	X	X	X
HDL-Cholesterol	X	X	X	X
LDL-Cholesterol	X	X	X	X
Systolic blood pressure	X	X	X	X
Diastolic blood pressure	X	X	X	X
CRP	X	X	X	X
Fetuin-A	X	X	X	X
Albumin-to-creatinine ratio (ACR),	X			
**Aim #4: Behavioral outcomes**				
Physical activity (actigraph, PPAQ)	X	X	X	X
Dietary intake (24-hr recalls)	X	X	X	X
**Censoring variables**				
Postpartumpregnancy test (urine)		X	X	X
75 g OGTT (postpartum)		X	X	X
**Eligibility variables**				
100 g OGTT (prenatal)	X			
**Covariates**				
Clinical characteristics of the current pregnancy	X			
Gestational weight gain	X			
Medical history	X			
Sociodemographic factors	X			
Smoking (Cotinine) and substance use	X	X	X	X
Postpartum factors (e.g., sleep, depression, breastfeeding)		X	X	X

Fasting biomarkers will be collected at baseline, and at 6-weeks, 6-months, and 1-year postpartum (Table 1). Postpartum biomarkers of insulin resistance will include glucose, insulin, HbA_1c_, leptin, TNF-α, HOMA, Area-Under-The-Glucose Curve (AUC), and adiponectin. Postpartum biomarkers of cardiovascular risk factors will include blood lipids, blood pressure, C-reactive protein (CRP), fetuin-A, and albumin-to-creatinine ratio (ACR). Postpartum diabetes screening will occur at each postpartum assessment and will follow the guidelines of the 2007 5th International Workshop Conference on GDM recommending a postpartum 75-g OGTT [49] using diagnostic criteria defined by the American Diabetes Association [31].

### Behavioral outcomes

Women will be provided with the ActiGraph GT3X-plus activity monitor to be worn on the wrist for 7-days at each of the assessment time periods (enrollment, 6-weeks, 6-months, and 12-months postpartum). Previous studies have reported reasonable validity under laboratory conditions among pregnant women [50], as well as under free-living conditions [51-53]. In addition, at each assessment time period, trained bilingual personnel blinded to the assigned intervention arm will conduct three unannounced 24-hr dietary recalls over a two-week period and administer the Pregnancy Physical Activity Questionnaire (PPAQ) [54]. The PPAQ is a semi-quantitative questionnaire which evaluates participation in household/caregiving, occupational, sports/exercise, and transportation activities.

### Covariates

Clinical characteristics of the current pregnancy and medical history will be abstracted from the pregnancy medical record (Table 1). Weight is measured prospectively at each prenatal visit and postpartum weight will be measured during assessment visits by trained study staff to the nearest 0.1 kg on accurately calibrated standard clinical scales using a standardized protocol. Gestational age will be based upon the best clinical estimate as recorded in the medical record. Adherence with gestational weight gain guidelines will be calculated by comparing the observed weight gain with the 2009 IOM Guidelines [27]. We will also collect gestational age at the time of GDM screen; degree of abnormality on glucose tolerance testing during pregnancy; treatment for glucose abnormality during pregnancy (e.g., diet, oral hypoglycemic agents and/or insulin); pregnancy complications; and birth outcomes.

Sociodemographic factors will be collected via self-report at enrollment. Acculturation will be assessed via the Psychological Acculturation Scale [55], language preference, and generation in the U.S. Smoking will also be assessed via the biomarker cotinine. Postpartum factors will be collected via self-report at each postpartum assessment and will include: The Pittsburgh Sleep Quality Index (PSQI) [56], the Edinburgh Postpartum Depression Scale [57,58], and the Infant Feeding Questionnaire [59].

### Data analysis

Primary analyses will evaluate differences in the change from 6 weeks postpartum to 12 months postpartum between the groups (an intent to treat analysis) in postpartum weight loss (aim #1), biomarkers of insulin resistance (aim #2), and biomarkers of cardiovascular disease risk (aim #3). We will use mixed models with random subject effects, including a common mean at baseline for the treatment groups, a period effect, and an intervention by period interaction [60]. The mixed model analysis will enable inclusion of time varying covariates such as breastfeeding behaviors, depression, and sleep, which may vary between baseline and follow-up for individuals. Equivalence of treatment groups will be assessed by comparing distributions of the potential confounders across groups. In addition, we will investigate established risk factors for type 2 diabetes as potential confounders or effect modifiers. Important potential effect modifiers include prepregnancy BMI, and breastfeeding behaviors. Mixed model analyses will also be used to account for the repeated measures of exercise and diet and will be used to evaluate adoption and maintenance of behavior change (aim #4) in the Lifestyle Intervention group.

### Power and sample size

The study is powered to detect mean differences in change in outcome variables from baseline in the Lifestyle Intervention as compared to the Health and Wellness (control) Intervention that are equivalent to 0.35 to 0.40 standard deviations, or a “small-medium” effect size [61], at 80% power at a 0.05 level of significance. A total of 256 women are required (128 participants per study arm). With an expected attrition rate of 15%, 300 eligible women will be recruited.

## Discussion

This trial is innovative in testing a prenatal and postpartum lifestyle intervention designed to reduce risk factors for type 2 diabetes and cardiovascular disease among high-risk Hispanic women. Changes in lifestyle risk factors (e.g., postpartum weight loss through reduction in energy intake and increased physical activity) among women who experienced abnormal glucose tolerance in pregnancy may reduce risk factors for type 2 diabetes and cardiovascular disease. Guidelines for the management of the elevated risk of diabetes among women with glucose abnormalities during pregnancy have lagged behind its recognition [62]. Women receive closer medical attention during the prenatal and postpartum periods than at other times in their adult lives, and are often highly motivated to improve their health to benefit themselves and their children. This postpartum lifestyle intervention capitalizes upon this teachable moment, and extends prenatal randomized trials conducted by our research team and others which utilized exercise and/or diet to reduce the risk of excessive gestational weight gain and gestational diabetes mellitus [36,63].

The low-income Hispanic population targeted by this study will include Spanish speakers (~25%), a population who have, overall, been excluded from research studies [64] or are underrepresented in research even when recruitment goals include Hispanics [65] despite the greater health challenges they face. The trial includes novel materials developed specifically for this group, thus increasing its innovation.

Investigation of which types of programs benefit postpartum women and identification of women at particularly high-risk are needed to increase the effectiveness of any prevention efforts. The public health impact of such lifestyle modifications is likely to be greatest in ethnic groups, such as Hispanics, with consistently high rates of obesity, diabetes, and the highest rates of sedentary behavior. This randomised controlled trial uses a high-reach, low-cost strategy, which can readily be translated into clinical practice in underserved and minority populations.

## Competing interests

The authors declare that they have no competing interests.

## Authors’ contributions

BHM, MCR, KLT, SJH, PP, BB, TAMS, CGS, JEM, and GM have made substantial contributions to the conception and design of the study, have been involved in revising the manuscript critically for important intellectual content; and have given final approval of the version to be published. LCT conceived of the study, made substantial contributions to conception and design of the study, drafted the manuscript, has been involved in revising the manuscript critically for important intellectual content, and has given final approval of the version to be published.

## Pre-publication history

The pre-publication history for this paper can be accessed here:

http://www.biomedcentral.com/1471-2393/14/100/prepub
